# The burden of birth control: a narrative review on the mood-related side effects of hormonal contraception

**DOI:** 10.1192/j.eurpsy.2025.2396

**Published:** 2025-08-26

**Authors:** B. P. Brás, J. F. Silva, F. Caldas, H. S. Almeida

**Affiliations:** 1 Unidade Local de Saúde do Santo António, Porto, Portugal

## Abstract

**Introduction:**

Hormonal contraception (HC) is widely used by females worldwide. Sex hormones – oestrogen and progesterone – affect the central nervous system’s function, structure, and neurotransmission, modulating emotional and behavioural responses. The use of HC, by introducing exogenous hormones and modulating the internal production of sex hormones, may be associated with mood changes and depressive symptoms. These symptoms are commonly reported by females taking HC and are one of the most frequent reasons for its discontinuation.

**Objectives:**

To explore the relationship between HC and depressive symptoms in females of reproductive age, with a focus on clinical implications.

**Methods:**

A narrative literature review was conducted using the *PubMed*® database with the search query: “(Hormonal Contraception) AND ((Mood) OR (depression))”. Studies published in the last 20 years were included.

**Results:**

Recent studies have shown an association between HC use and depression. The relationship between HC and mood changes is complex and influenced by various factors, including the type of HC, dosage, patients’ psychiatric history, and psychosocial factors. The link between HC and depression seems to be related to the dosage and type of progestogen. Also, the use of progestins with androgenic activity, such as levonorgestrel, may carry a higher risk of deleterious mood changes. Adolescent females, those with a personal or family history of mood disorders, females with premenstrual dysphoric disorder or premenstrual syndrome, and those who have experienced adverse mood effects with previous use of HC are more predisposed to developing depressive symptoms related to HC. HC is also associated with sexual dysfunction and an increased risk of suicide and suicide attempt. On the other hand, continuous use of HC may provide relief from depressive symptoms in females with premenstrual dysphoric disorder by stabilising fluctuations in hormone levels. Limited evidence suggests that HC use among females with depressive or bipolar disorders is not associated with a worse clinical course compared to the use of non-hormonal methods.

**Conclusions:**

The mechanism underlying how HC influences mood remains poorly understood. In clinical practice, the effects of HC on mood seem to be most relevant in selected subsets of females. Most females using HC demonstrate no effect or a beneficial effect on mood, with a low incidence of adverse effects. The risk of adverse mood effects should not preclude the prescription of HC. Mental health risk factors, as well as any newly present or ongoing mental health symptoms, should be considered when initiating and reviewing HC in the management and treatment of female patients.

**Disclosure of Interest:**

None Declared

